# Does Modern Lifestyle Favor Neuroimmunometabolic Changes? A Path to Obesity

**DOI:** 10.3389/fnut.2021.705545

**Published:** 2021-09-21

**Authors:** Camila Guazzelli Marques, Marcus V. L. dos Santos Quaresma, Fernanda Patti Nakamoto, Ana Carolina Oumatu Magalhães, Glaice Aparecida Lucin, Ronaldo Vagner Thomatieli-Santos

**Affiliations:** ^1^Programa de Pós-graduação em Psicobiologia, Departamento de Psicobiologia, Universidade Federal de São Paulo, São Paulo, Brazil; ^2^Departamento de Nutrição, Centro Universitário São Camilo, São Paulo, Brazil; ^3^Departamento de Biociências, Universidade Federal de São Paulo, Santos, Brazil

**Keywords:** obesity, lifestyle, neuroendocrine-control, hypothalamic inflammation, food intake

## Abstract

Factors linked to modern lifestyles, such as physical inactivity, Western diet, and poor sleep quality have been identified as key contributors to the positive energy balance (PEB). PEB rises adipose tissue hypertrophy and dysfunction over the years, affecting cells and tissues that are metabolically critical for energy homeostasis regulation, especially skeletal muscle, hypothalamic-pituitary-adrenal axis, and gut microbiota. It is known that the interaction among lifestyle factors and tissue metabolic dysfunction increases low-grade chronic systemic inflammation, leading to insulin resistance and other adverse metabolic disorders. Although immunometabolic mechanisms are widely discussed in obesity, neuroimmunoendocrine pathways have gained notoriety, as a link to neuroinflammation and central nervous system disorders. Hypothalamic inflammation has been associated with food intake dysregulation, which comprises homeostatic and non-homeostatic mechanisms, promoting eating behavior changes related to the obesity prevalence. The purpose of this review is to provide an updated and integrated perspective on the effects of Western diet, sleep debt, and physical exercise on the regulation of energy homeostasis and low-grade chronic systemic inflammation. Subsequently, we discuss the intersection between systemic inflammation and neuroinflammation and how it can contribute to energy imbalance, favoring obesity. Finally, we propose a model of interactions between systemic inflammation and neuroinflammation, providing new insights into preventive and therapeutic targets for obesity.

## Introduction

Obesity is defined as an excessive accumulation of adipose tissue, commonly related to low-grade chronic systemic inflammation (LGCSI) (1, 2). Both obesity and LGCSI are considered crucial independent risk factors for several chronic non-communicable diseases, including cardiovascular diseases, type 2 diabetes mellitus, some types of cancer, musculoskeletal disorders, and other clinical conditions (1, 3), reducing life expectancy (3, 4). Moreover, mid-life obesity is a significant risk factor for developing Alzheimer's disease and vascular dementia in later life (5, 6). Early to mid-adulthood obesity may have a detrimental impact on cognitive functioning, due to decreased brain volume (7), gray matter atrophy in the temporal, frontal and occipital cortices, hippocampus, thalamus and midbrain (8, 9), and reduced integrity of white matter throughout the brain (10).

Obesity incidence and prevalence increased rapidly in the last 50 years, currently reaching pandemic proportions (1, 3). According to the latest data published by the World Health Organization (WHO), in 2016, more than 650 million adults were obese, representing 13% of the world population (11). The epidemiological perspectives are even more alarming (1, 12). According to the projections made by Kelly and collaborators, in 2008, 1.12 billion people will be obese in 2030 (13).

The nature of obesity is attributed to the chronic positive energy balance (PEB: caloric intake greater than daily energy expenditure) (3, 14). However, weight gain is a complex and multifactorial phenomenon. Obesity is determined by a matrix of genetic, epigenetic, psychological, and lifestyle factors, which interact with the physical sociocultural environment and suffer its interference, influencing several physiological mediators responsible for food intake and energy expenditure (1, 14).

Swinburn et al. (15) suggest that the high prevalence of obesity is the result of human response to the obesogenic the obesogenic environment, evidenced in most regions of the world in the last five decades by nutritional, demographic, and socioeconomic changes associated with the processes of urbanization and globalization (16).

In recent years, rapid changes occurred in the global food system and in the frequency of physical activity practice, factors that interact with human behavior and lifestyle, leading to PEB (3, 15, 16). Besides food intake, one of the main changes observed was the increase in supply and accessibility to high energy-rich foods, which are more palatable, processed, and ultra-processed (15). These foods contain high amounts of sugars, trans and saturated fats, dietary salt, food additives, while present low contents of fiber, carbohydrates accessible to the gut microbiota (CAM), polyunsaturated fats, vitamins, minerals, and bioactive compounds (16, 17).

Simultaneously, sedentary behavior is highly prevalent, especially in high-income countries, due to urbanization and technological advancement (15, 18). Currently, three key domains favor sedentary lifestyle among adults: work, transport, and leisure (19). The term sedentary behavior includes time spent sitting with low energy expenditure levels (19). For example, watching television, reading, driving, and most of the work done at an office desk (20). In addition to the increasing levels of sedentary behavior, there is a concern about the low prevalence of regular physical exercise (PE), mainly moderate to vigorous, among several populations in the world (20). According to the WHO, 1 in 4 adults does not reach recommended levels of PE in the world (21). Health benefits of physical activity are well-established, including its contribution to a healthy body mass maintenance (22).

Although less debated and neglected by society in recent years, sleep duration and quality have also been negatively affected (23). Short sleep duration is associated with metabolic, immunological, and behavioral changes that compromise health status and predispose to weight gain (24–27). Systematic reviews and meta-analyses have shown an association between short sleep duration and obesity (25, 28, 29).

However, there is a segmented discussion about the influence of these three factors on body mass gain and obesity. We believe that common mechanisms are shared and overlapped, especially involving systemic inflammation and neuroinflammation, which may explain why isolated interventions against obesity are ineffective.

The purpose of this review is to provide an updated and integrated perspective on the effects of the Western diet (WD), sleep debt, and PE on the regulation of energy homeostasis and low-grade chronic systemic inflammation. Subsequently, we discuss the intersection between systemic inflammation and neuroinflammation and how it can contribute to energy imbalance, favoring obesity. Finally, we propose a model of interactions between systemic inflammation and neuroinflammation, providing new insights into preventive and therapeutic targets for obesity.

## Energy Balance

Individually and combined, WD, sleep debt, and sedentary behavior can promote a favorable environment for fat mass increase and metabolic dysregulation. Several mechanisms have been proposed in attempt to explain why this triad can increase body mass. Beyond physiological, biochemical, and inflammatory aspects, the main factor involved is the chronic PEB.

Energy balance (EB) is based on energy intake, expenditure, and storage (30, 31). The balance between energy intake and expenditure promotes the energy homeostasis maintenance and both overnutrition and malnutrition result from positive and negative EB, respectively (30). However, the simplistic and linear conception between energy intake and expenditure is not enough to explain weight gain (32). The concept of “energy in” and “energy out” has been refuted, since numerous factors can modify substrate availability, utilization, and storage (33, 34). However, the new models proposed still lack robust evidence (35).

Furthermore, the static view of EB (“eat less” vs. “exercise more”) can be challenged once weight gain, *per se*, increases resting energy expenditure (REE), total energy expenditure (TEE), as well as energy cost due to the need to move a larger body (36). In contrast, weight loss is accompanied by a reduction in REE, non-resting energy expenditure (nREE), and TEE. Weight loss-related low energy expenditure is known as metabolic adaptation or adaptive thermogenesis, described as a barrier to successful weight loss maintenance (37). However, recent evidence suggests that metabolic adaptation is irrelevant, and it is possibly not associated with weight regain (38, 39). For this reason, EB is considered to be highly complex, influenced by several factors, some of which are poorly understood. Chronic PEB, resulting from high energy intake and low energy expenditure, increases fat storage (triglycerides) in the white adipose tissue (WAT). WAT hypertrophy and lower adipogenesis promote an inflammatory state triggered by activation of several immunological pathways (40–42). Gregor and Hotamisligil (43) proposed the term “metainflammation” for the inflammatory state mediated by the excess of nutrients in obesity. Metainflammation is mainly mediated by macrophages and occurs in several tissues, such as large intestine (gut microbiota), liver, skeletal muscle, and adipose tissue. Macrophages adopt different activation states (i.e., M1 and M2 polarization) depending on the energy environment (44, 45). High energy levels promote M1 polarization and inflammatory cytokines production (44, 45). It is worth noting that, especially in microglia, the concept of M1 and M2 polarization, by IFN- γ (interferon-gama) and interleukin (IL)-4 secretion, respectively, is questionable because this phenomenon has not been established by research findings, bringing up more complex insight about mechanisms related to microglial activation (46).

Nutrition contribution to metainflammation depends on macronutrient type, its quantity and effect on different immune cells, and its interaction with the gut microbiota, a vital place for inflammatory changes (47).

## Inflammation

Inflammation is characterized by a sophisticated process of pathogens elimination added to tissue repair and recovery, involving immune and other types of cells. According to the degree and extent of the inflammatory process (local or systemic), several metabolic and neuroendocrine changes can occur (48, 49). The inflammatory response is traditionally initiated by infections, resulting from the interaction between pattern-recognizing receptors expressed in cells of the innate immune system and pathogen-associated molecular patterns (PAMPs); likewise, damage-associated molecular patterns (DAMPs) trigger inflammatory response during conditions of physical stress, chemical stress, or through harmful metabolites such as biglycan, fibrinogen, uric acid, mtDNA, among others (50). The shift from a transient to a chronic inflammatory state affects immune tolerance, leading to modifications in the functions of tissues and organs, increasing chronic non-communicable risk for diseases (50). This chronic effect is known as LGCSI. In the absence of PAMPs, DAMPs play a role in triggering the inflammatory process. LGCSI is increased with aging, obesity, chronic infections (i.e., HIV), presence of metabolic diseases such as type 2 diabetes mellitus, non-alcoholic fatty liver, and pollutants (50). Moreover, LGCSI is directly regulated by factors such as diet, sleep, and sedentary lifestyle, in such a manner that this interaction is crucial for the development of several tissue modifications.

## Neuroinflammation

**Figure 1:**
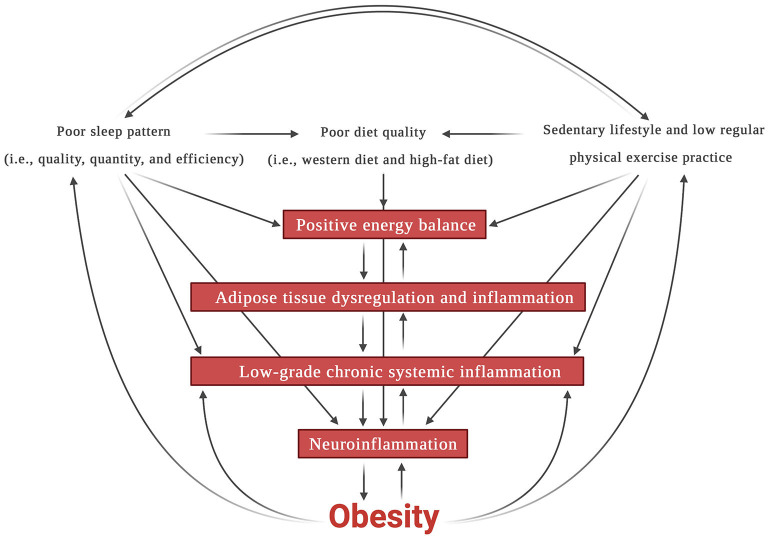
Interconnections among sleep, diet, sedentarism, inflammation, and obesity. Created with BioRender.com.

In recent years, the relationship between inflammation and the brain, mainly the hypothalamus region, has gained attention, being recognized as neuroinflammation. Interestingly, LGCSI, neuroinflammation, and obesity share similar factors and mechanisms (Figure 1).

The immune system and brain are linked by nerve fibers, soluble mediators (i.e., cytokines), and leukocyte traffic (26). Cytokines are proteins that regulate the inflammatory and immune response, produced in response to several pathogens and other antigens. Cytokines act in autocrine, paracrine, and endocrine fashion. Several functions are related to cytokines, such as class switching in B cells, T helper cells differentiation into Th-1 or Th-2, among other subsets, and phagocytes activation (51).

Likewise, immune cells can recognize neurotransmitters and neuropeptides, such as catecholamines and neuropeptide Y. Still, leukocytes can synthesize and release several neuronal messengers. Other cells such as microglia and astrocytes could act as immune cells, mediating the inflammatory process in the brain. Both are defined as non-neuronal cells, recognized as determinants of energy homeostasis. Microglia-related effects appear to be influenced by microglia location on different anatomical regions (i.e., basal ganglia, substantia nigra, etc.). Furthermore, microglia profile is primarily dependent on their interaction with several cell types (i.e., neurons and astrocytes, etc.) by membrane-bound pattern recognition receptors (PRRs), such as PAMPs, or by cellular damage (DAMPs) (52).

Astrocytes are defined as a subtype of glial cells, which comprise most cells in the central nervous system (CNS). Several functions are related to astrocytes (i.e., neuronal survival, Blood-Brain Barrier (BBB) regulation, synapses formation, neuroprotective effect by excessive neurotransmitters removal, secretion of trophic factors, etc.). However, they do not conduct electrical signals, but play important roles in numerous brain-related functions, being the target of treatment of CNS diseases (53). Additionally, astrocytes interact with numerous brain cells during neuroinflammation. It is believed that a bidirectional communication between astrocytes and microglia modulates CNS-related inflammation by multiple cytokines and inflammatory mediators (53, 54). Interestingly, microglia appears to play a dual role in sustaining BBB integrity during systemic inflammation. However, more studies are needed, in order to fully elucidate these mechanisms (55).

This crosstalk between the immune system and the brain is influenced by factors commonly discussed for health maintenance, such as diet, exercise, and sleep (26). For instance, in recent years, high energy intake has gained attention as a mediator of meta-inflammation and this pathway seems to play a crucial role in LGCSI and neuroinflammation. Therefore, EB has a prominent role in neuroimmunoendocrine regulation.

The EB regulation occurs through a complex and improved bidirectional communication between peripheral organs and hypothalamus (56, 57). Hypothalamus is composed of different nuclei with specific functions, but the arcuate nucleus of the mediobasal hypothalamus is recognized for playing a pivotal role in regulating energy homeostasis (58). Interestingly, hypothalamus inflammation appears to precede LGCSI in obesity (56, 59–62). Several studies showed that diet could affect inflammatory biomarkers related to neuroinflammation.

Thaler et al. (63) showed an increase in the inflammatory response in rodents's hypothalamus after 1–3 days of a high-fat diet (HFD; 60% kcal of fat; 5.24 kcal/g) differently from the inflammatory response observed in peripheral tissues. Besides, inflammatory biomarkers suggestive of neuronal injury, microglial accumulation, and reactive gliosis in the hypothalamic arcuate nucleus were observed in the 1st week of HFD. These authors also identified that hypothalamic inflammatory responses temporarily decreased to baseline values, between the 7th and 14th days, with a subsequent increase in inflammatory markers on the 28th day. These data suggest that sustained HFD could promote neuroinflammation, despite the attempt of the brain, through neuroprotective mechanisms, to mitigate inflammation. The relevance of these findings is also attributed to the increase in inflammation regardless of the change in the animals' body weight in the 1st days of the protocol. Other studies have observed that the increase in energy intake has determinant effects on hypothalamic inflammation (58) and, added to the energy supply, it is believed that saturated fatty acids (SFA) play a vital role as inflammatory triggers (60). SFAs seem to be the primary inflammatory trigger on the hypothalamus by interacting with toll-like receptor 4 (TLR-4) (56).

In 2005, De Souza et al. (59) published the first study that demonstrated positive association between HFD, obesity, and hypothalamic inflammation. The authors observed high expression of c-Jun N-terminal kinase (JNK) and NF-kB (factor nuclear kappa B), leading to an increase of TNF-alpha (tumor necrosis factor alpha), IL-6, and IL-1beta levels, impairing insulin and leptin signaling after 16 weeks of HFD.

Later, other studies demonstrated similar results (60, 61). Other than triggering inflammatory pathways through TLR-4, SFAs could promote hypothalamic inflammation by stressing the endoplasmic reticulum and protein kinase CQ (PKCQ), which may negatively affect the neurons involved in satiety response (64). Moreover, leptin also affects microglial activation, increasing the inflammatory state (56).

Activated glial cells (microglia and astrocytes) produce inflammatory chemokines and cytokines, affecting neuropeptides involved in EB regulation (56). Inflammatory cytokines secreted by microglia induce the expression of CX3C-1 (fractalcin) by neurons, which potentiates and maintains hypothalamic inflammation (65). Although little investigated, the increased expression of CCL5 [Chemokine (C-C motif) ligand 5] seems to promote the exacerbated activation of hypothalamic MCH (melanin-concentrating hormone) neurons (56).

## Diet, Sleep, and Physical Exercise Influence on Inflammation

### Western Diet

The term WD derives from one of the changes in lifestyle that has occurred in the last decades in Westernized societies: an increase in the consumption of processed foods, “fast food,” convenience products, snacks, and sugary soft drinks, while fibers, vitamins, and minerals are lacking. It is important to notice that WD differs from HFD, specially concerning the content and profile of fat. The majority of preclinical reports use HFD composed of rodent chow plus 45–65% of calories as saturated fat, whereas modern WDs contain about 33% of calories as fat, and only about one-third of that is saturated (66, 67). In fact, fat type and amount were shown to differentially affect the development of adiposity in different obese rodent strains (68) and unevenly affect fatty acid metabolism and contractile function in the heart of Wistar rats (69). At the same time, both WD and HFD may contribute to increase inflammation.

WD promotes weight gain due to (i) chronic PEB and (ii) nutrients that act as antigens, triggering immune activation. The high amount of sugar and SFA significantly increases energy intake, leading to increased fat mass. Moreover, the composition of WD can increase the odds of metabolic disorders development, such as insulin resistance and poor lipid profile, promoting an unregulated metabolic environment that will maximize adipose tissue-related disorders (70, 71).

Shively et al. (66, 72), in a randomized, pre-clinical study, submitted non-human primates to WD or Mediterranean diet (MD). After 2.5 years, the authors found that WD, added to increased fat mass, promoted insulin resistance and hepatic steatosis, while MD improved lipid profile. This data is essential to nutrition science, since intervention studies in humans are methodologically complex. More recently, WD has also been described as a dietary pattern capable of modifying the inflammatory environment (73). The relationship between LGCSI, dietary pattern, and worsened health status are widely discussed nowadays, especially because several nutrients and dietetic compounds trigger an immune response (73).

Specific components of WD (i.e., fat, sugar, and dietary salt) stimulated inflammatory process in studies conducted in animal models (74) and humans (75, 76). For instance, high intake of monosaccharides (i.e., glucose and fructose) leads to hyperglycemia and advanced glycation products (AGEs) synthesis, recognized by their receptors (rAGE), activating the NF-kB pathway, kinases, and reactive oxygen species (ROS) (77). SFAs stimulate TLR-2 and TLR-4, producing inflammatory cytokines (77). Other mechanisms, mediated by fatty acids, are also proposed to explain the increase in the inflammatory process, such as ceramide biosynthesis and NLRP3 (NOD-, LRR- and pyrin domain-containing protein 3) inflammasome activation (77). Although recent studies suggest that SFAs do not physically interact with TLR-4, it is believed that this type of fat may act on the stability of this receptor, also contributing to increase the inflammatory process (78).

The impact of WD on the inflammatory process is not limited to sugar and SFA. This dietary pattern lacks CAM, fruits, and vegetables (79). Moreover, it is common to verify high consumption of dietary salt, alcoholic beverages, and ultra-processed foods (79). This combination maximizes the inflammatory process through several pathways. Poor CAM diet can impair the composition of gut microbiota and its activity, increasing intestinal permeability and the leaky gut process (80). Poor CAM diet also decreases the amount of mucus, lowering the intestinal physical barrier against opportunistic antigens (81–83). The leaky gut leads to increased bacterial fragments (i.e., lipopolysaccharide; LPS) in the bloodstream. LPS, through specific receptors interacts with several cell types, including immune cells, increasing the production of inflammatory cytokines (84).

Moreover, dietary salt, found in large quantities in WD, can influence the differentiation of CD4^+^ *naive* T lymphocytes into T helper (Th)−17, which is capable of increasing inflammation, as well as reducing the expression and activity of anti-inflammatory cells such as regulatory T lymphocytes (85). High dietary salt intake has been associated with several adverse outcomes that run through inflammation (86). Other evidences indicate that zinc deficiency (87), magnesium (88), and omega-3 essential lipids (precursor of resolvins, maresins, and protectins) (89, 90) also contribute to the imbalance between pro- and anti-inflammatory mediators.

#### Western Diet, Adipose Tissue, and Inflammation

In addition to the factors mentioned above, it is crucial to consider that the effect of WD on inflammation leads to body composition changes. *Per se*, fat mass gain promotes LGCSI. Adipose tissue, recognized as an endocrine organ, plays a vital role in the inflammatory balance, producing and releasing several inflammatory and anti-inflammatory cytokines, known as adipokines (91).

Both adipocyte hypertrophy and dysregulation increase pro-inflammatory mediators (i.e., IL-6, IL-1β, and TNF-alpha) and decrease anti-inflammatory mediators (i.e., adiponectin and omethine-1) (92). WAT hypertrophy mediated by chronic PEB may promote macrophage phenotypic changes (polarization of M1 macrophages), increasing the production of inflammatory cytokines, such as TNF-alpha, IL-6, and chemokines such as monocyte chemoattractant protein-1 (MCP-1), whose function is to increase the recruitment of monocytes to adipose tissue. In contrast, by reducing levels of adiponectin, mitigation mechanisms of inflammation are minimized (93). Adiponectin may reduce the inflammatory process mediated by LPS in macrophages by inhibiting the NF-kB pathway, reducing M1 and increasing M2 polarization (93). Moreover, large adipocytes lose its ability to store triacylglycerol and display impaired energy expenditure. WAT dysregulation creates a vicious and inflammatory cycle. TNF-α increases lipolysis of triacylglycerol into fat-free acids (FFA), which have affinity for TLR-4 on the surface of both adipocytes and macrophages, increasing the inflammatory process *via* NF-kB (91). Despite the attempt of the adipose tissue to accommodate excessive consumed energy, its capacity is limited, resulting in activation of apoptosis mechanisms. For example, the switch from lipogenesis to lipolysis, mediated by insulin resistance and lower glucose uptake, promotes adipocyte death (94). Thus, high FFA circulation leads to fat accumulation in other organs (i.e., muscle, liver, pancreas, heart, and kidney), known as ectopic fat accumulation, causing peripheral metabolic tissue dysregulation along with increased LGCSI (95).

Finally, recent evidence suggests that WD effects, which modulate inflammatory mediator's gene expression in adipose tissue, are dependent on the gut microbiota (96). Therefore, even though mechanisms have not been completely elucidated so far, the relationship between nutrients, adipose tissue, and inflammation has been identified as an essential element for creating interventions capable of mitigating inflammatory-related diseases.

### Short Sleep Duration

Sleep consists of a natural state necessary for life, being characterized by high neurophysiological activity (97). In studies with humans and rodents, the sleep debt (deprivation, restriction, or fragmentation) caused broad physiological changes that, in this review, will be directed to immune activation, inflammation, body composition, and obesity.

Van Leeuwen et al. (98) found that sleep restriction (4 h of sleep per night) for five nights promoted lymphocytes immune activation and inflammatory cytokines (i.e., IL-6, IL-1B, IL-17) synthesis. The authors also noted that inflammatory mediators remained elevated 2 days after the whole period of sleep restriction (98). Other published studies corroborate these findings, reinforcing the effect of sleep debt on the inflammatory process (99, 100). Short sleep duration and poor sleep quality increase the activity of the stress response system: the sympathetic nervous system (SNS) and the Hypothalamus-Pituitary-Adrenal (HPA) axis. The SNS is responsible for the release of norepinephrine by nerve fibers and adrenaline by the adrenal gland, both of which can activate beta-2 adrenergic receptors in leukocytes, increasing inflammatory cytokines' expression. Regarding cortisol, despite being a natural anti-inflammatory hormone, sleep restriction persistently maintains the activation of HPA axis, promoting cortisol resistance, reducing its effect in suppressing inflammatory cytokines. The relationship between sleep and inflammation appears to be bidirectional, creating a vicious cycle (101).

Global sleep loss, as a result of a 24/7 society, suggests that people who are chronically sleep-deprived experience LGCSI, leading to increased fat mass and metabolic disorders (102). Some studies suggest that sleep debt is associated with an increase in inflammatory mediators (103, 104) and obesity (105). Zhou et al. (106) studied Obstructive Sleep Apnea Syndrome (OSAS), a pathological sleep disruption model, and verified the negative effect of patients sleep debt on inflammatory mediators and, consequently, on health outcomes.

From the bidirectional relationship between obesity and inflammation, sleep seems to have a crucial role as its mediator (102). In the last two decades, studies with rodents and humans have shown that sleep debt promotes several metabolic dysregulations (107, 108), mediated, at least in part, by an increase in the inflammatory process (104), insulin resistance (109), worsening of lipid profile (110), physical inactivity (111), and increased food intake (112). Therefore, it is understood that sleep debt is inserted in a matrix of factors that interact with each other. Nevertheless, it is not simple to define cause and consequence.

The relationship between sleep debt, inflammatory pathways, and EB has been identified as an essential obesity mediator (113). The most consolidated theoretical assumption is based on the effect of sleep debt on the imbalance between leptin and ghrelin (homeostatic regulation of food control) (114, 115) and the deregulation of the hedonic system (non-homeostatic food control) (116). This deregulation promotes eating behavior changes (i.e., skipping meals, snacking, and mealtimes irregularity) (113), supporting weight gain.

Previous studies identified that sleep debt could increase ghrelin levels and reduce leptin levels, modifying the activity of the satiety center in the ventromedial and arcuate nucleus of the hypothalamus (117, 118). Nedeltcheva et al. (119) identified that sleep debt (5.5 vs. 8.5 h) for 14 days increased carbohydrate intake after 7 p.m., mainly due to increased snack intake. St-Onge et al. (120) found that the short sleep time (4 h per night) for five nights increased energy and total fat intake, especially saturated fat. Sleep debt studies have also shown an increase in food craving, suggesting that EB changes can occur by eating behavior-related aspects (121, 122). These findings were confirmed in epidemiological studies (123, 124) and recent systematic reviews, which have shown that sleep debt promotes worse food choices (i.e., snacks, sugar, fat) (125, 126). Moreover, indirect pathways mediated by sleep debt can also affect food intake. Sleep debt elevates anxiety and depression symptoms, which modify food intake (127). Deregulation of these systems can lead to weight gain by high-energy intake. Besides, stress response systems (i.e., SNS and HPA axis) are activated in response to sleep debt, which can modify emotional aspects and the way of dealing with the stressful environment, leading to changes in eating behavior (128). Likewise, Choi et al. (129) found that sleep debt increases stress perception, a critical factor for high energy food intake, especially more palatable foods (i.e., sweet and fat-rich foods) promoting comfort and pleasure, known as “comfort food” (130).

Added to weight gain and overnutrition, which promote LGCSI, sleep debt increases processed food and WD nutrients (sugar and SFA) intake, maximizing the inflammatory process. Finally, sleep debt can promote a more sedentary lifestyle, decreasing TEE, PEB, and LGCSI state (131).

### Physical Exercise

Regular PE is a crucial factor for human health. Several mechanisms are proposed to explain PE-related health benefits. PE increases REE and TEE (132). Still, PE (especially endurance exercise) can promote muscle-related metabolic adaptations increasing FFA uptake and oxidation (133). Additionally, both acute and regular strength training affect energy expenditure. It is suggested that strength training acute increases in REE last 24-h after exercise and that chronic raises in REE occur due to skeletal muscle hypertrophy (134, 135). Muscle mass is one of the most variable components of fat-free mass (FFM), which represents ~22.5% of REE (136), reinforcing its importance for EB.

PE-related effects in mitochondrial biogenesis and gene expression of several proteins of lipid metabolism (i.e., lypolisis, FFA transportation, its musculoskeletal uptake, mitochondrial internalization, and beta-oxidation) are extensively well-described in the literature (133).

High muscle FFA oxidation capacity prevents accumulation of ceramides and other compounds related to insulin resistance and inflammatory state, especially in overnutrition situations (137). PE-related benefits are not limited to the factors mentioned above. More recently, researchers verified that PE can modify shape and activity of gut microbiota. Products of energy metabolism originating from the skeletal muscle (i.e., lactate) can reach the gastrointestinal tract and trigger positive gut microbiota changes (138).

Moderate-intensity PE improves immune function, increases antioxidant defense mechanisms, and decreases ROS production (139). PE is temporarily able to promote immune cells (i.e., lymphocytes) redistribution to peripheral tissues (i.e., lungs and intestines), increasing immunocompetence (140). Regular PE also promotes an anti-inflammatory environment, decreasing the odds of developing inflammatory-related diseases (139).

When stimulated by PE, skeletal muscle is recognized as an endocrine organ, synthesizing and releasing several cytokines, named myokines (i.e., irisin, IGF-1, FGF2, IL-3, IL-6, IL-8, IL-10, IL-15, IL-1ra) (141). Although the mechanisms are not entirely elucidated, these myokines play a pivotal role in inflammatory balance (142). For example, IL-6, a pleotropic hormone-like cytokine, influences fat and glucose metabolism during exercise, especially in glycogen-depleted conditions. Previous studies showed that IL-6 mediates glucose uptake through adenosine monophosphate kinase (AMPK). AMPK is an energy cell sensor that recognizes intracellular low-energy levels (143), improving lipolysis, glycogenolysis, gluconeogenesis, and fat and glucose oxidation (144, 145). IL-6 energy-regulated effect is essential to improve glycemia and lipid profile during exercise (146).

The main IL-6 anti-inflammatory effect is related to IL-10 increases. IL-6 triggers IL-10 production by immune cells, supporting an anti-inflammatory state (147, 148). Recently, Alizaei et al. (149) conducted a systematic review and meta-analysis and found that both aerobic and strength exercise can reduce inflammatory biomarkers (i.e., TNF-α and C-reactive protein) and increase IL-10. The increase in IL-10 occurs mainly in response to endurance exercises.

Moreover, IL-6 positive anti-inflammatory effects also appear to be related to WAT decrease. Recently, Wedell-Neergaard et al. (150) verified that IL-6 is indispensable to PE-related effect on visceral adipose tissue (VAT). These authors offered the subjects tocilizumab (IL-6 inhibitor) and placebo in a 12-week cycling exercise protocol. Exercise plus tocilizumab group did not reduce VAT, suggesting that VAT-lipolysis is IL-6 dependent.

Moreover, irisin, a hormone-like myokine, product of fibronectin type III domain-containing protein 5 (FNDC5) from skeletal muscle in response to PE, acts on WAT, mediating the browning process (151). Brown adipose tissue (BAT) increases energy expenditure, improves metabolic efficiency, and reduces insulin resistance (152, 153). Several studies reinforce the BAT role in health maintenance, improving several metabolic biomarkers. For example, BAT contributes to glucose and dietary fatty acids uptake; moreover, BAT activity appears to be protected against conditions linked to WAT excess (154).

Other mechanisms are described in attempt to explain the broad PE anti-inflammatory pathways, including (i) containment of the inflammatory process by reducing VAT, increasing circulating levels of adiponectin, reducing monocytes infiltration into adipose tissue, and diminishing macrophage polarization to M1 phenotype in WAT (155, 156); (ii) reduced expression of TLRs in immune cells and other tissues (i.e., adipose tissue); (iii) increased adrenaline, cortisol, growth hormone, and prolactin levels, which have an immunomodulatory role due to the ability to influence leukocyte functioning and traffic; (iv) elevation of catecholamines circulating level, inhibiting LPS-induced inflammatory effect (156). It is worth mentioning that Hill et al. (157) observed that different intensities of PE may elicit different cortisol responses (the threshold phenomenon). Moderate to high intensity exercise provoked increases in circulating cortisol levels (specially glycogen depletion and IL-6 rise), while low intensity exercise (40% maximal oxygen uptake) did not result in significant increases in cortisol levels. Moreover, regular PE could decrease inflammatory trigger molecules release in the resting state and in response to exercise (i.e., IL-6). This training adaptation effect could explain, at least in part, the lowest levels of anti-inflammatory agents (i.e., cortisol) in the resting state (158).

Finally, although the mechanism is not fully elucidated, several studies have identified that skeletal muscle is one of the main organs responsible for resolving inflammation. Furthermore, several cross-sectional and longitudinal studies verified negative association between muscle mass, morbidity, and mortality (159–162).

## Inflammation, Neuroinflammation, and Obesity: Relationship, Between Diet, Sleep, and Physical Exercise

WD and sleep debt promote an inflammatory state, as described above. In contrast, PE can counterbalance LGCSI. Despite mechanistic similarities, the interaction between these factors is little explored, especially in neuroinflammation. Thus, we explored WD, sleep debt, and PE-related effect on neuroinflammation and how they affect obesity. LGCSI, neuroinflammation, and obesity share several similar pathways, but they are usually discussed separately. It reinforces our idea of discussing these factors jointly.

Considering the pivotal role of inflammatory mediators on hypothalamus EB deregulation, we discussed that obesity and neuroinflammation appear to share a bidirectional relationship. Beyond hormones, the hypothalamus is also sensitive to nutrients. Leptin and insulin, which play essential metabolic roles, reach the hypothalamus, activating sacietogenic neuropeptides. However, in obesity, the hypothalamus is resistant to these hormones, justifying the increase in energy intake (163, 164). Neuroinflammation leads to insulin and leptin resistance in the hypothalamus (165, 166). Additionally, high leptin levels also contribute to maintaining the inflammatory process (167). Recent studies conducted with rodents suggest that reduced neuroinflammation increases the sensitivity to leptin in the hypothalamus, reducing food intake and weight gain (168). In the last years, several studies verified the effect of neuroinflammation on food intake regulation pathways and how HFD can increase inflammatory mediators in the brain. Zhang et al. (58) found that high expression of inhibitor of nuclear factor kappa-**β** (IKK-**β**) in the hypothalamus affects food intake. The authors also verified that IKK-**β** suppression mitigates weight gain and leptin/insulin resistance in rats submitted to HFD. Likewise, Posey et al. (61) found that, after administering an IKK-**β** inhibitor in rats, insulin sensitivity in the hypothalamus increased, reducing food intake and body weight during HFD.

Kim et al. (169) found that HFD-fed mice and their offspring showed increased BBB disruption, probably caused by changes in the tanycyte population (a specialized ependymal cell in the brain) and expression of transporters. Additionally, astrocytes and microglia, which are important for maintaining BBB integrity, supporting neuronal metabolism, and preventing/responding to local tissue injury, have increased activation in the hypothalamus of rodents and humans with HFD consumption (170). Another previous study also reported the increase in BBB permeability in response to HFD (171). Since BBB is a critical regulatory interface in the communication between the peripheral tissues and the CNS (172), disruptions could increase inflammatory mediators access to the CNS. Still, Jin et al. (173) verified that astrocyte-specific Myeloid differentiation primary response 88 (MyD88) knockout mice were resistant to HFD-induced obesity and to leptin action, suggesting that the deletion of hypothalamic inflammatory pathways (i.e., MyD88) can mitigate the adverse changes caused by HFD (174).

These classic studies reinforce the role of inflammation in increasing body weight and metabolic perturbations. Beyond SFAs-related effects on neuroinflammation, stress and anxiety responsiveness appear to be mediated by SFA. Both stress and anxiety trigger high-energy-density foods intake (175, 176). This relationship contributes to a better understanding of this bidirectional mechanism between inflammation and energy intake (177).

Negative effects of WD on gut microbiota, intestinal permeability, LPS-mediated immune activation, and systemic inflammation have been considered as critical pathways for microglia activation and induction of neuroinflammation (178). Microglia, responsible for cytokines release in the brain (similar to macrophages), is responsive to LPS by expressing receptors for PAMPs, increasing the M1 phenotype, and producing inflammatory cytokines (179, 180). Observational studies that associate dietary patterns and brain outcomes have increased in recent years (181, 182). Dietary patterns with higher amounts of nutrients obtained from animal sources (animal protein, cobalamin, cholesterol, and omega-6) increase circulating inflammatory biomarkers. In contrast, plant-based dietary patterns decrease inflammatory markers, mainly in people with a less healthy lifestyle and sleep disorders (183). These data suggest, at least in part, the WD pivotal food role on inflammation, especially in individuals who are more prone to systemic inflammation, usually with a sedentary lifestyle and poor sleep quality.

Previous studies verified high levels of inflammatory biomarkers after sleep debt protocols (184). Interestingly, it is suggested that the increased inflammatory state induced by poor sleep also occurs by changes in gut microbiota and adipose tissue (185). The synergy between the immunological system and sleep is complex and it has several mechanisms to explain this bidirectional relationship (26). Sleep debt effects on neuroinflammation have been described previously (186). More recently, Ho et al. (187) verified that sleep fragmentation protocol (18 h of sleep fragmentation per every 24-h period) plus HFD promote microglial activation. Three days of exposure to sleep fragmentation or HFD increased Iba-1-ir (Ionized calcium binding adaptor molecule 1 immunoreactivity) in the arcuate nucleus and the ventromedial hypothalamus. Still, after 9 days, Iba-1-ir remained elevated in the arcuate nucleus in sleep fragmentation plus HFD group, suggesting an interactive effect of both factors.

CNS permissiveness also increases in response to sleep debt. Studies have shown that sleep disturbances increase BBB permeability (188, 189). Its high permeability, also favored by LGCSI, may increase immune-activating substances (i.e., PAMPs and DAMPs) passage and inflammatory cytokines release in the CNS (190). Moreover, sleep debt (18 h of fragmentation for 21 days) triggered neuroinflammation, promoting anxiogenic response, such as high-energy low-quality food intake (191). HFD and sleep debt share similar neuroinflammatory mediating mechanisms, leading to insulin/leptin resistance and anxiety, crucial for weight gain and obesity (192). Amiri and Behnezhad (193) conducted a systematic review and meta-analysis and identified 10 and 30% increased anxiety symptoms odds in overweight and obese subjects, respectively. High anxiety levels also occur in sleep debt (194). This relationship is even more interesting when obesity and sleep loss increase emotional eating (dimension of eating behavior stimulated by anxiety), an essential obesity-related factor (195).

Chronic cortisol exposure as a result of sleep debt could increase the mesolimbic reward system, increasing palatable food intake (196). Cortisol also plays a pivotal role in leptin and ghrelin signaling, which may affect EB (197, 198). Also, cortisol reactivity is a crucial factor that modulates eating behavior. For instance, Herhaus et al. (199) observed that obese subjects with high cortisol reactivity demonstrated a significantly higher food intake than subjects with low cortisol reactivity. Interestingly, they did not verify this effect in lean subjects (199). Finally, cortisol increases blood glucose and adiposity, modulating metabolic pathways related to energy expenditure, energy intake, and body composition (197).

Considering PE in the discussion of neuroinflammation is essential. In response to muscular contractions, myocytes produce and release several molecules, named myokines (200). IL-6 myokine upregulates the expressions of anti-inflammatory cytokine IL-10 and the levels of IL-1 receptor antagonist (IL-1Ra) (45). It has been shown that long-term moderate-intensity PE can increase the production and secretion of IL-10 in the skeletal muscles (201, 202). When IL-10 interacts with its receptor on microglia, it enhances the suppressor of cytokine signaling (SOCS) 3, an inhibitor of cytokine-induced signaling responses, resulting in inhibition of microglial activation, thus acting against the inflammatory state (203).

Microglia polarization on different phenotypes is mainly responsible for the hypothalamus inflammatory state. Lower microglia IL-10 levels favors polarization to M1 phenotype, increasing inflammation (180). Other studies reinforce the pivotal IL-10-blunted inflammatory effect in microglia (180). IL-10 could interact with astrocytes and neurons in the CNS, decreasing inflammatory mediators (180). PE can also stimulate the expression of IL-1Ra in the CNS, which has a higher affinity for the IL-1R than IL-1α or IL-1β. Blocking the binding of IL-1 to its receptor interrupts the pro-inflammatory IL-1 signaling cascade and related microglial activity (204, 205). Therefore, exercise can upregulate the expression of anti-inflammatory cytokines and inhibit microglial activation.

Although less elucidated to the present day, PE can also contribute to neuroinflammation control by the kynurenine (KYN) pathway. The inflammatory state can modify tryptophan metabolism, leading to the formation of KYN. KYN can take two paths, kynurenic acid (KYNA) or quinolinic acid (QUIN). While KYNA has positive effects, such as inflammation counterbalance, QUIN increases the oxidative process and neurotoxicity. The inflammatory environment appears to be an essential mediator for conversion of KYN to QUIN since high TNF-alpha levels increase QUIN production (206). On the other hand, PE promotes the conversion of KYN to KYNA, with neuroprotective-related effects.

**Figure 2:**
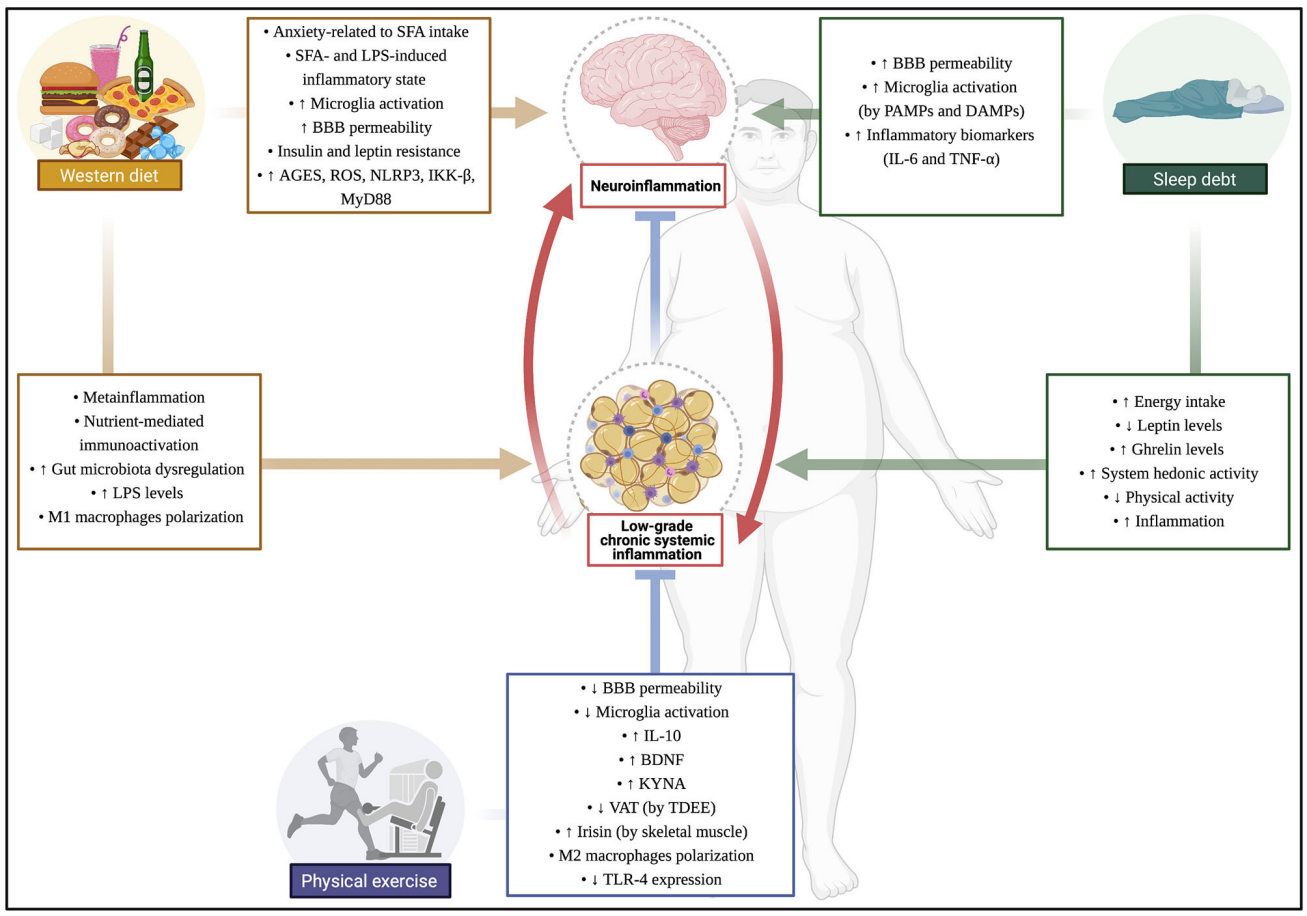
Consequences of Western diet, sleep debt, and physical exercise on inflammation and obesity. Created with BioRender.com. Legend: ↑, high/increase; ↓, low/decrease; SFA, saturated fatty acids; LPS, lipopolysaccharide; AGES, advanced glycation products; ROS, reactive oxygen species; NLRP3, NOD-, LRR- and pyrin domain-containing protein 3; IKKβ, inhibitor of nuclear factor kappa-B kinase subunit beta; MyD88, Myeloid differentiation primary response 88; BBB, Brain-Blood-Barrier; PAMPs, pathogen-associated molecular patterns; DAMPs, damage-associated molecular patterns; IL-6, Interleukin-6; TNF-α, tumor necrosis factor-alpha; IL-10, Interleukin-10; BDNF, Brain-derived Neurotrophic Factor; KYNA, kynurenic acid; VAT, visceral adipose tissue; TDEE, total daily energy expenditure; TLR-4, toll-like receptor 4.

PE also improves BBB permeability, increasing gene expression of tight junctions (206). Moreover, the role of Brain-derived Neurotrophic Factor (BDNF) in inflammatory control has been discussed (207). While WD and sleep debt can decrease BDNF levels and increase oxidative stress (208), BDNF release by PE contributes to inflammatory control, although more robust evidence is needed (209). Low BDNF levels appear to be related to hyperphagia, weight gain, and obesity. Still, some studies verified that BDNF administration restores regular food intake (210), reinforcing that BDNF can affect energy homeostasis and body composition; but, for now, despite the motivating studies, the evidences are still contradictory (211) (Figure 2).

## Future Directions and Clinical Perspective

The sleep-diet-exercise triad, therefore, should be analyzed in conjunction in future research. Few studies have evaluated these three factors simultaneously and observed obesity- or CNS-related changes in humans. One of them was conducted by Wickham et al. (212). They named this triad “The Big Three Health Behaviors” and investigated the differential and higher-order associations between sleep, physical activity, and dietary factors as predictors of mental health and well-being in young adults. Although their findings suggest that sleep quality was the strongest predictor of depressive symptoms and well-being, they state that physical activity and diet are secondary, but still significant factors. Accordingly, Martinez et al. (213). examined the interplay between sleep, diet, and physical activity on obesity-related parameters, with more robust methods, in a 2-year cohort study of Mexican American children. They found that longer sleep duration was associated with lower body weight in baseline, 1- and 2-year follow-up. Also, children with higher physical activity levels had lower body weight. Moreover, children with higher energy intake had higher body weight at a 2-year follow-up. Their findings suggest that longer sleep duration plays a consistent and protective role against childhood obesity and that moderate-to-vigorous physical activity and health energy intake are important independent factors for obtaining a healthy weight. It is expected that new evidence become available soon, due to advances in techniques for the objective analysis of energy expenditure, sleep, dietary patterns, PE practice, body composition, and brain-related parameters. Still, the progress of omic sciences (i.e., genomics, transcriptomics, proteomics, or metabolomics) has contributed to a deeper understanding of the mechanisms involved in the chronic inflammatory process (i.e., gut microbiota, gut-derived metabolites, cellular residues with inflammatory trigger features, etc.), its consequences, and its main predictors. Clinicians must understand that this “blend factor effect” is always present in the context of obesity and should employ this more comprehensive interpretation to perform enhanced interventions. Clinical practices which do not contemplate all the factors may lead to deficient preventive actions or poor treatment outcomes.

## Conclusion

WD, sleep debt, and PE regulate the inflammatory state. WD and sleep debt similarly maximize the inflammatory mediators, energy intake, weight gain, and obesity. On the other hand, PE increases energy expenditure and metabolic efficiency and counterbalances inflammatory mediators, promoting weight gain and obesity resistance. Systemic inflammation and neuroinflammation in obesity are complex responses and share multifactorial features, hindering the establishment of just one mechanism. Future studies should consider that multi-interaction factors contribute to the inflammatory state, making way for further discussions on more strategies capable of regulating the inflammatory process.

## Author Contributions

RT-S and CM generated the research question. CM, MS, FN, AM, and GL performed the literature search. CM, MS, and FN wrote the manuscript. RT-S and FN critically reviewed the manuscript. All authors contributed and approved the final version for submission.

## Funding

This work was supported by Fundação de Amparo à Pesquisa do Estado de São Paulo (FAPESP) FAPESP: 2019/22524-8.

## Conflict of Interest

The authors declare that the research was conducted in the absence of any commercial or financial relationships that could be construed as a potential conflict of interest.

## Publisher's Note

All claims expressed in this article are solely those of the authors and do not necessarily represent those of their affiliated organizations, or those of the publisher, the editors and the reviewers. Any product that may be evaluated in this article, or claim that may be made by its manufacturer, is not guaranteed or endorsed by the publisher.
